# Supplementary material for: Parylene C as a Multipurpose Material for Electronics and Microfluidics
Source: Polymers (Basel). 2023 May 12;15(10):2277. doi: 10.3390/polym15102277 (PMC10224276; doi:10.3390/polym15102277)
Supplement: Supplementary file 1 [file polymers-15-02277-s001.zip › polymers-2333685-supplementary.pdf]

polymers

MDPI

# Supplementary Materials

# Parylene C as a Multipurpose Material for Electronics and Microfluidics

**Beatriz J. Coelho [1,2], Joana V. Pinto [1,*], Jorge Martins [1], Ana Rovisco [1], Pedro Barquinha [1], Elvira Fortunato [1], Pedro V. Baptista [2], Rodrigo Martins [1] and Rui Igreja [1,*]**

1 CENIMAT|i3N, Department of Materials Science, NOVA School of Science and Technology, Campus de Caparica, NOVA University of Lisbon and CEMOP/UNINOVA, 2829-516 Caparica, Portugal; bj.coelho@campus.fct.unl.pt (B.J.C.); jds.martins@campus.fct.unl.pt (J.M.); a.rovisco@fct.unl.pt (A.R.); pmcb@fct.unl.pt (P.B.); emf@fct.unl.pt (E.F.); rfpm@fct.unl.pt (R.M.)
2 UCIBIO, I4HB, Department of Life Sciences, NOVA School of Science and Technology, Campus de Caparica, NOVA University of Lisbon, 2829-516 Caparica, Portugal; pmvb@fct.unl.pt
* Correspondence: jdvp@fct.unl.pt (J.V.P.); rni@fct.unl.pt (R.I.)

## 1. Capacitance of the MIM structures

Figure S1 illustrates the capacitance (C) and capacitance per unit area (C/A) at 5 kHz measured for MIM structures produced with Parylene C and PTFE as the dielectric layer, prior to any stimulus.

(a) Capacitance (C) for single-layered Parylene C

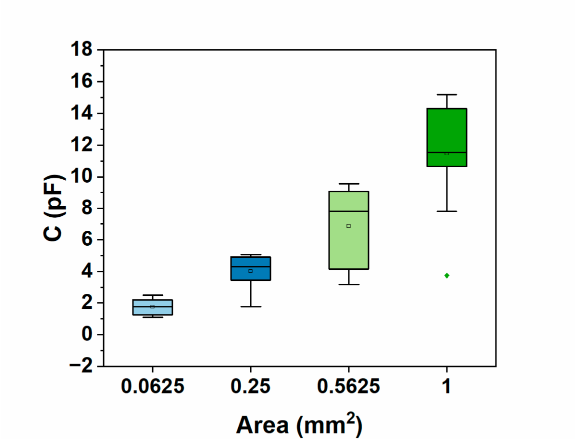


(b) Capacitance (C) for double-layered Parylene C

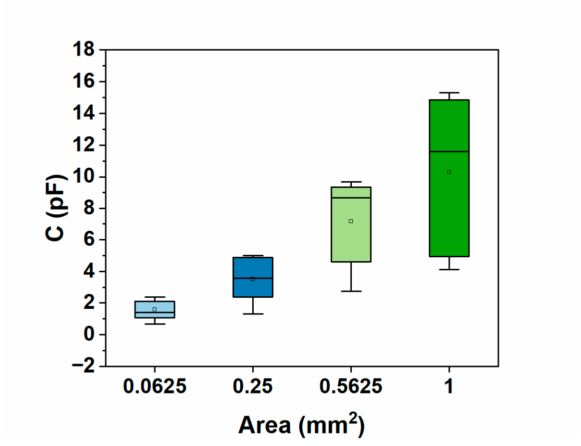


(c) Capacitance per unit area (C/A) for single-layered Parylene C

(d) Capacitance per unit area (C/A) for double-layered Parylene C

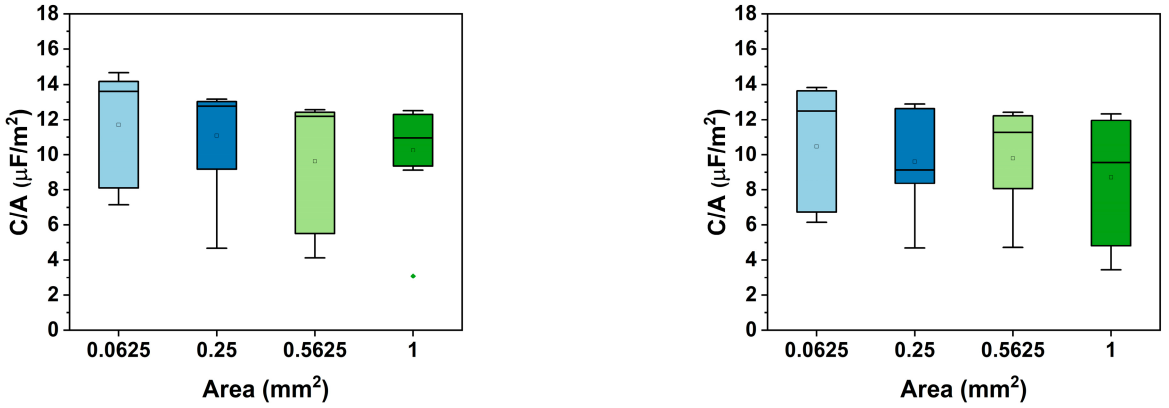


**Figure S1.** Capacitance measured for the MIM structures used in this work, prior to any stimulus. (**a**) Capacitance measured for devices with single-layered Parylene C; (**b**) Capacitance measured for devices with double-layered Parylene C; (**c**) Capacitance per unit area measured for devices with single-layered Parylene C; (**d**) Capacitance per unit area measured for devices with double-layered Parylene C.

As can be observed in Figure S1, for both single- (a) and double-layered (b) Parylene C, the absolute capacitance increases with the MIM area, as expected. There is, however, a slightly greater dispersion of the capacitance values for double-layered Parylene C, which could be due to interfacial phenomena between the two layers. As for the capacitance per unit area for single-layered Parylene C, some abnormal values are present, as may be inferred from the greater distances between maximum and minimum value points in the box charts. Nevertheless, for all the areas studied, the median value is quite stable, ranging from 11.0 μF/m² to 13.6 μF/m². Regarding double-layered Parylene C, abnormal values are also present, however the median value remains fairly stable across MIM areas, despite presenting a moderately higher dispersion (9.1 μF/m² to 12.5 μF/m²). Once again, this moderate dispersion could be due to interfacial phenomena between the two layers, not accounted for with the normalization per area.

## 2. Relative permittivity of the MIM structures

Similarly to the capacitance (see Supplementary Materials section 1), the relative permittivity ($\varepsilon_r$) was determined for the MIM structures at 5 kHz, prior to any stimulus being applied – Figure S2.

(a) Relative permittivity ($\varepsilon_r$) for single-layered Parylene C

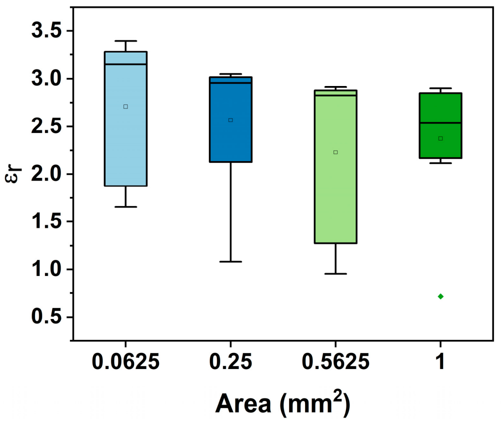


(b) Relative permittivity ($\varepsilon_r$) for double-layered Parylene C

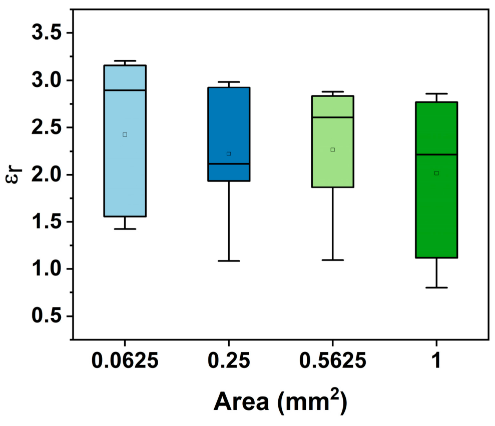

**Figure S2.** Relative permittivity for MIM structures produced with PTFE and Parylene C deposited in single (**a**) or double layer (**b**).

As can be seen on Figure S2a, the median relative permittivity of single-layered Parylene C MIM structures is approximately 3.0, which is consistent with the $\varepsilon_r$ reported for Parylene C only (see Table S1). This also illustrates the reduced influence that the PTFE layer has on the overall dielectric layer, which would be expected considering the small thickness of this layer (50 nm) as compared to the Parylene C thickness (2 µm). Nevertheless, for double-layered Parylene C, a greater variation in the permittivity results is observed, which may be attributed to the interface between the two Parylene C layers.

**Table S1.** Relative permittivity measured for Parylene C, according to different suppliers.

| Relative Permittivity | Frequency | Reference |
|---|---|---|
| 2.65 | 1 kHz | 1 |
| 3.10 | 1 kHz | 2 |
| 3.10 | 1 kHz | 3 (material used in this study) |

**3. Loss tangent of the MIM structures**

Figure S3 illustrates the loss tangent for the MIM structures assembled within the scope of this work.

(a) Loss tangent for single-layered Parylene C

(b) Loss tangent for double-layered Parylene C

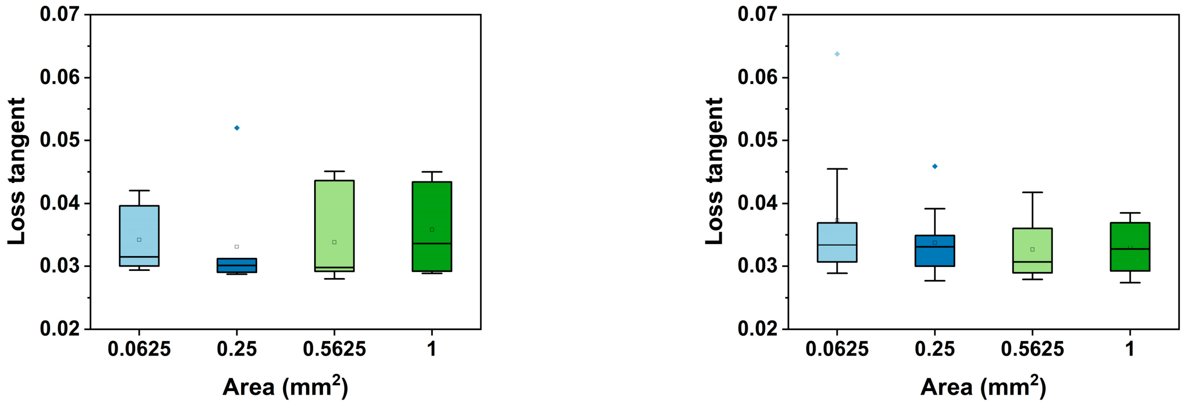


**Figure S3.** Loss tangent for the MIM structures, composed of either single- (**a**) or double-layered (**b**) Parylene C, measured at 5 kHz.

As can be seen from Figure S3, the median loss tangent for both single- and double-layered Parylene C is quite low, indicating that the dielectric layer composed of PTFE and Parylene C presents low energy dissipation.

Figure S4 presents the frequency dependance of the loss tangent for MIM structures with different thicknesses, for single-layered Parylene C, and a fixed MIM area of 1 mm$^2$ (area of overlapping top and back contacts, as depicted in Figure 1c of the main text).

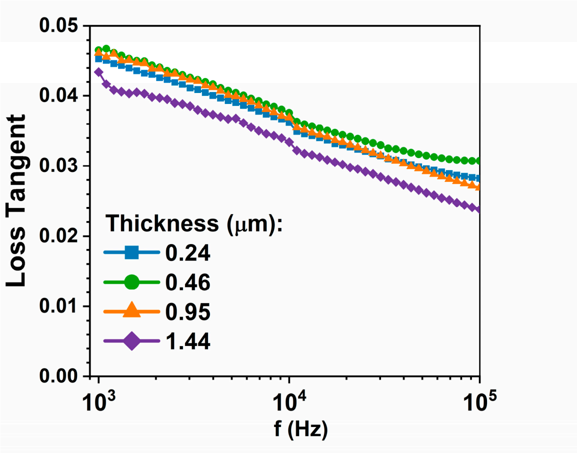


**Figure S4.** Loss tangent for MIM structures with different thicknesses (single-layered Parylene C). Device area is 1 $mm^2$.

## 4. Parylene C-only in comparison to Parylene C-Tantalum Pentoxide as dielectric layer for digital microfluidic devices

Another strategy under development by our group is the use of two different materials comprising the dielectric layer in digital microfluidic (DMF) devices, in order to increase the robustness of such layer for long reaction times. Specifically, 715 nm of Parylene C was coupled with 260 nm of $Ta_2O_5$ (tantalum pentoxide) to form the dielectric layer. Figure S5 illustrates the behavior of the capacitance of the dielectric layer with frequency, as well as droplet speeds tested on our DMF devices with the fore-mentioned dielectric layer.

(a) (b)

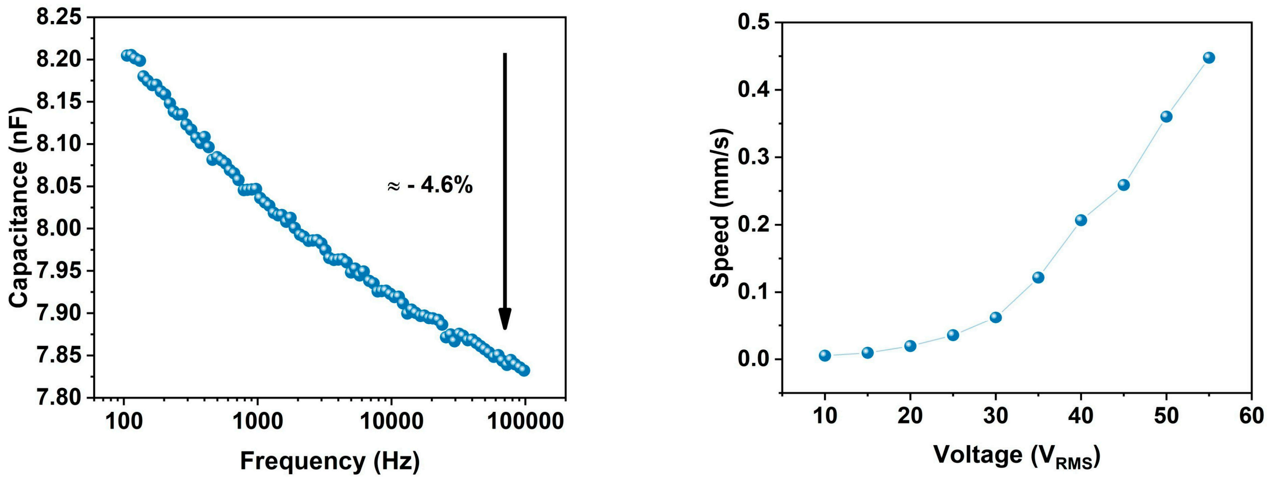


**Figure S5.** Measured properties of DMF devices containing a $Ta_2O_5$-Parylene C dielectric layer. (**a**) Capacitance of the dielectric layer over frequency. (**b**) Measured speed of an actuated droplet (3 M of an aqueous sodium chloride solution) moving over a DMF device, for several actuation voltages (constant actuation frequency, 5 kHz).

As depicted on Figure S5a, the capacitance of the $Ta_2O_5$-Parylene C dielectric layer remains stable over a wide scope of frequencies, ranging from 100 Hz to 100 kHz. Capacitance decreases merely 4.6% over said frequency range, suggesting a good stability of the dielectric film. Particularly, at 5 kHz, the typical actuation frequency for the DMF devices developed within the group, the capacitance reaches 7.95 nF as opposed to 11.5 pF achieved with single-layered Parylene C or 11.6 pF (median capacitances) achieved with

double-layered Parylene C (both with a total thickness of 2 μm), considering the same area of 1 mm$^2$.

## 5. Cross sections of the bottom plate of a digital microfluidic device

Figure S6 illustrates several images of the bottom plate of a DMF device, acquired by SEM, evidencing all the layers composing this plate. Please note that DMF devices were frozen in liquid nitrogen and broken afterwards, so that all layers were visible. Cutting the substrates without the liquid nitrogen treatment would lead to a complete cover of the chromium layer by Parylene C.

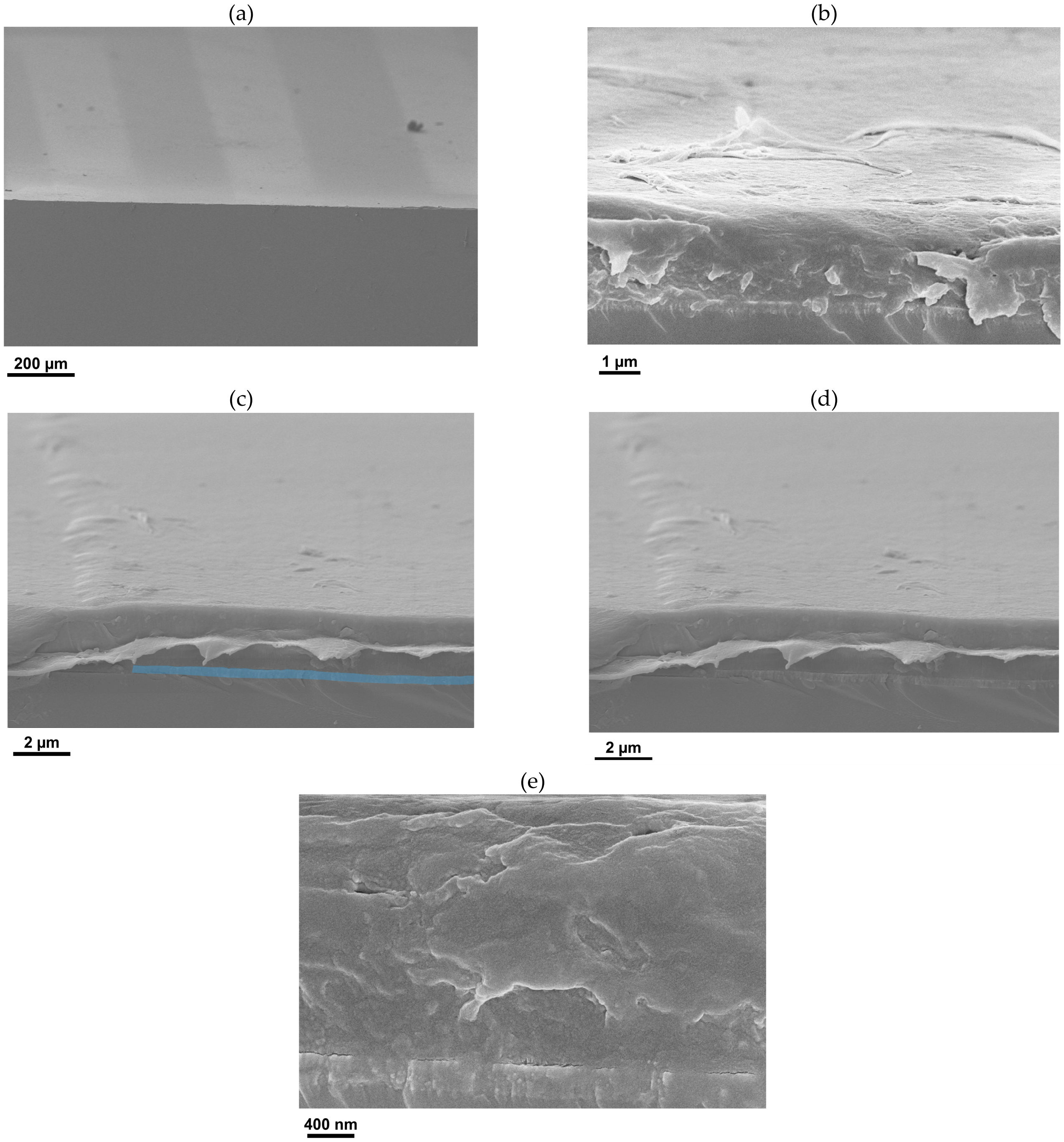


**Figure S6.** SEM-acquired cross-sectional views of the bottom plate of a DMF devices, evidencing all the composing layers. (**a**) Top view of the DMF bottom plate, where chromium connector lines are visible below the Parylene C and PTFE layers, in a light gray color. (**b**) Surface of the DMF bottom plate, where the PTFE layer is visible as two small wrinkles bending over the Parylene C layer. (**c**) Detail of the cross section of a chromium connector line, with chromium highlighted in blue. It is

also visible that the layers on top of the chromium suffer an increase in height, which follows the length of the connector line. (**d**) Representation of Figure 5c without the blue filter for the chromium connector line, where the interface between chromium and the glass substrate, as well as the interface between chromium and Parylene C, are clearly visible. (**e**) Detail of the interface between Parylene C (on top) and chromium (at the bottom).

## References


1. VSI Parylene. Parylene Properties. https://vsiparylene.com/parylene-properties/ (2022).
2. Advanced Coatings. Parylene C Specifications. https://www.advancedcoating.com/typical-specification-parylene-c (2022).
3. Specialty Coating Systems. *SCS Parylene Properties*. https://scscoatings.com/technical-library/ (2018).
